# Enhanced Development of Azoxymethane-Induced Colonic Preneoplastic Lesions in Hypertensive Rats

**DOI:** 10.3390/ijms140714700

**Published:** 2013-07-15

**Authors:** Takahiro Kochi, Masahito Shimizu, Tomohiko Ohno, Atsushi Baba, Takafumi Sumi, Masaya Kubota, Yohei Shirakami, Hisashi Tsurumi, Takuji Tanaka, Hisataka Moriwaki

**Affiliations:** 1 Department of Internal Medicine, Gifu University Graduate School of Medicine, Gifu 501-1194, Japan; E-Mails: kottii924@yahoo.co.jp (T.K.); tomohikooh@hotmail.com (T.O.); babagif@yahoo.co.jp (A.B.); sumieron@gmail.com (T.S.); kubota-gif@umin.ac.jp (M.K.); shirakamiyy@yahoo.co.jp (Y.S.); htsuru@gifu-u.ac.jp (H.T.); hmori@gifu-u.ac.jp (H.M.); 2 Department of Tumor Pathology, Gifu University Graduate School of Medicine, Gifu 501-1194, Japan; E-Mail: takutt@toukaisaibou.co.jp

**Keywords:** hypertension, colon carcinogenesis, oxidative stress, inflammation, angiotensin-II

## Abstract

Metabolic syndrome is associated with an increased risk of colorectal cancer. This study investigated the impact of hypertension, a component of metabolic syndrome, on azoxymethane (AOM)-induced colorectal carcinogenesis using SHRSP/Izm (SHRSP) non-diabetic/hypertensive rats and SHRSP.Z-*Lepr^fa^*/IzmDmcr (SHRSP-ZF) diabetic/hypertensive rats. Male 6-week-old SHRSP, SHRSP-ZF, and control non-diabetic/normotensive Wister Kyoto/Izm (WKY) rats were given 2 weekly intraperitoneal injections of AOM (20 mg/kg body weight). Two weeks after the last injection of AOM, the SHRSP and SHRSP-ZF rats became hypertensive compared to the control WKY rats. Serum levels of angiotensin-II, the active product of the renin-angiotensin system, were elevated in both SHRSP and SHRSP-ZF rats, but only the SHRSP-ZF rats developed insulin resistance, dyslipidemia, and hyperleptinemia and exhibited an increase in adipose tissue. The development of AOM-induced colonic preneoplastic lesions and aberrant crypts foci, was significantly accelerated in both SHRSP and SHRSP-ZF hypertensive rats, compared to WKY normotensive rats. Furthermore, induction of oxidative stress and exacerbation of inflammation were observed in the colonic mucosa and systemically in SHRSP and SHRSP-ZF rats. Our findings suggest that hypertension plays a role in the early stage of colorectal carcinogenesis by inducing oxidative stress and chronic inflammation, which might be associated with activation of the renin-angiotensin system.

## 1. Introduction

Obesity-related systemic metabolic dysfunctions such as diabetes mellitus, hypertension, and dyslipidemia are collectively known as metabolic syndrome (Mets) and pose serious health problems throughout the world [1,2]. In addition to the morbidity associated with these metabolic disorders, recent studies have revealed that Mets is linked to an increased risk of cancer in several organ sites including the colorectum [3–8]. Several pathophysiological mechanisms for this association have been described, including the emergence of insulin resistance, the state of chronic inflammation, induction of oxidative stress, and occurrence of adipokine imbalance [5,6]. In particular, diabetes is closely associated with the development of colorectal cancer (CRC) as obesity is the main determinant of insulin resistance and hyperinsulinemia [7].

Epidemiological studies have also revealed that hypertension may increase the risk of CRC [3,4]. The renin-angiotensin system is a key regulator of cardiovascular function, and its activation is involved in the etiology of Mets, especially hypertension [9]. There is increasing evidence that the renin-angiotensin system may have paracrine and autocrine functions with regard to tissue oxidative stress and chronic inflammation, as well as cellular proliferation and apoptosis [10–14]. In addition, dysregulation of the renin-angiotensin system has been reported to occur in human malignancies and has been shown to influence cancer cell migration, invasion, and metastasis, all of which are associated with a poor prognosis [10,11,14]. However, the precise mechanisms by which hypertension plays a role in the early stage of colorectal carcinogenesis remain unclear.

The stroke-prone spontaneously hypertensive rat (SHRSP) is a substrain of the spontaneously hypertensive rat (SHR), crossed and further inbred with selected offspring of parents that died of stroke. The SHRSP rats have a higher blood pressure than SHR rats and readily develop apoplexy. The crossing of SHRSP rats with Zucker Fatty (ZF) rats produces SHRSP.Z-*Lepr^fa^*/IzmDmcr (SHRSP-ZF) rats, which develop hypertension and become obese due to the leptin receptor *OB-rb* gene mutation carried by ZF rats [15]. SHRSP-ZF rats therefore exhibit a phenotype similar to human Mets and thus may be a useful model to investigate the molecular mechanisms underlying hypertension-related metabolic abnormalities [15,16]. However, colorectal carcinogenesis models using these rats have not been established.

The objective of this study was to determine the susceptibility of SHRSP-ZF and SHRSP rats to azoxymethane (AOM)-induced colorectal carcinogenesis and the utility of these rats as models for Mets, in particular, as models for hypertension-associated colorectal carcinogenesis, that appropriately reflect the pathological conditions of human Mets.

## 2. Results and Discussion

### 2.1. General Observations

Table 1 compares the mean body weights, adipose tissue weights, and blood pressures (systolic and diastolic) at the end of the study (10 weeks of age) between 3 groups (Group 1, Wister Kyoto/Izm [WKY] rats; Group 2, SHRSP rats; and Group 3, SHRSP-ZF rats) that received AOM. The mean body weights of WKY (*p* < 0.001) and SHRSP-ZF (*p* < 0.05) rats were significantly higher than that of SHRSP rats, but there was no significant difference between the WKY and SHRSP-ZF rats. There was a significant increase in the mean adipose tissue weights in SHRSP-ZF rats compared to WKY (*p* < 0.001) and SHRSP rats (*p* < 0.05). The systolic and diastolic blood pressures of SHRSP and SHRSP-ZF rats were markedly higher than those of WKY rats (*p* < 0.001). However, compared to SHRSP-ZF rats, SHRSP rats had marked hypertension (*p* < 0.05).

### 2.2. Serum Parameters of the Experimental Rats

As shown in Table 2, the serum levels of glucose and insulin significantly increased, but the value of QUICKI, a useful index of insulin sensitivity [17], was decreased in SHRSP-ZF rats compared to WKY and SHRSP rats (*p* < 0.05). The serum levels of leptin, non-esterified fatty acid (NEFA), and triglycerides in SHRSP-ZF rats were also significantly higher than those in WKY and SHRSP rats (*p* < 0.05). These findings suggest that SHRSP-ZF rats developed insulin resistance, hyperleptinemia, and dyslipidemia, all of which are frequently observed in human Mets patients. There were no significant differences in these serum components between WKY and SHRSP rats. The SHRSP and SHRSP-ZF rats did, however, have significantly elevated levels of serum angiotensin-II (AT-II), the active product of the renin-angiotensin system [18], compared to the WKY rats (*p* < 0.05), indicating that the renin-angiotensin system is activated in these hypertensive rats.

### 2.3. Development of Colonic Preneoplastic Lesions

**Figure 1:**
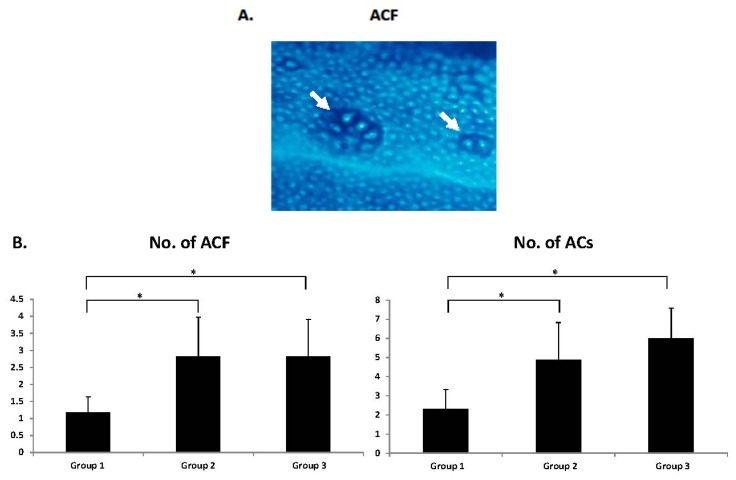
ACF developed in the SHRSP, SHRSP-ZF, and WKY rats that received AOM. (**A**) Representative morphology of ACF (arrows) induced by AOM stained with methylene blue in Group 2. Magnification, 40×; (**B**) Average number of ACF and ACs (/cm^2^). Group 1: WKY rats, Group 2: SHRSP rats, and Group 3: SHRSP-ZF rats. The values are expressed as mean ± SD. * *p* < 0.05.

Irrespective of the rat strain, aberrant crypt foci (ACF) (Figure 1A) were observed in the colon of all rats given AOM at the end of the study. However, the number and size (aberrant crypts [ACs] per cm^2^) of ACF were significantly greater in both the SHRSP and SHRSP-ZF rats than in the WKY rats (Figure 1B; *p* < 0.05). There was no significant difference in the development of ACF between SHRSP and SHRSP-ZF rats, indicating that hypertension, a common pathophysiological characteristic of these rats, plays a critical role in accelerating the development of colonic preneoplastic lesions.

### 2.4. Systemic Oxidative Stress and Colonic Epithelial Expression of GPx and CAT mRNA

**Figure 2:**
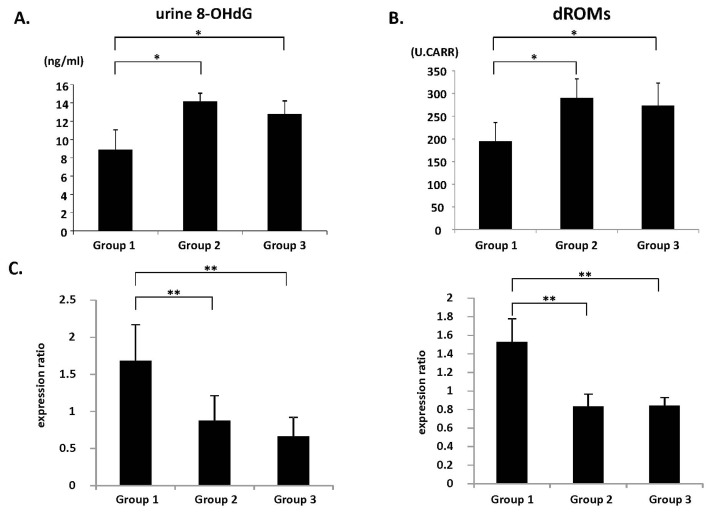
Measures of oxidative stress and antioxidant biomarkers’ expression. (**A**) Urine 8-OHdG levels were measured by enzyme immunoassay; (**B**) Hydroperoxide levels in the serum were determined by the d-ROM test; (**C**) The expression levels of *GPx* and *CAT* mRNA in the colonic epithelium were examined by quantitative real-time RT-PCR using specific primers. The values are expressed as mean ± SD. * *p* < 0.01, ** *p* < 0.01.

Oxidative stress is implicated in Mets and colorectal tumorigenesis [5]. Therefore, the levels of oxidative stress and antioxidant biomarkers in the experimental rats were assessed. Compared to the WKY rats, the SHRSP and SHRSP-ZF rats had significantly increased levels of urine 8-hydroxy-2′-deoxyguanosine (8-OHdG) (Figure 2A; *p* < 0.01), a marker of DNA damage induced by oxidative stress, and serum derivatives of reactive oxygen metabolites (d-ROM) (Figure 2B; *p* < 0.01), which reflects serum hydroperoxide levels. However, the SHRSP and SHRSP-ZF rats also had reduced expression levels of glutathione peroxidase (*GPx*) and catalase (*CAT*) mRNA, which encode antioxidant enzymes, in the colonic epithelium (Figure 2C; *p* < 0.05). These findings suggest that systemic oxidative stress is increased, whereas colonic antioxidant activity is decreased, in both SHRSP and SHRSP-ZF hypertensive rats.

### 2.5. Serum and Colonic Epithelial Expression of Inflammatory Markers

**Figure 3:**
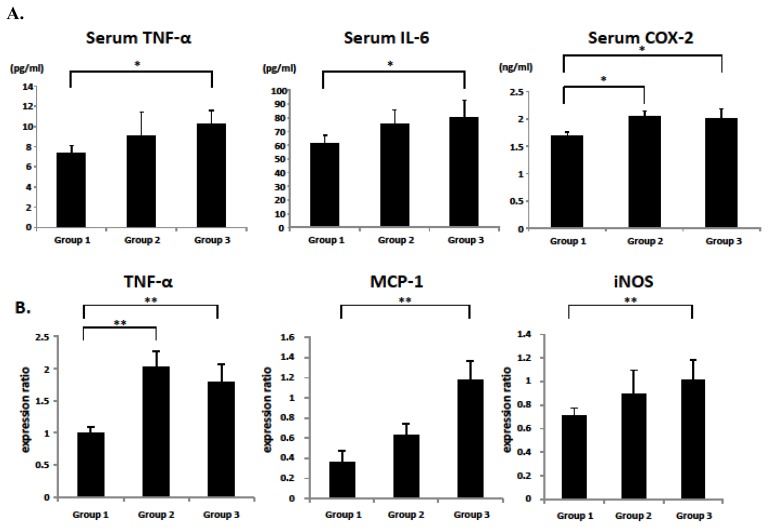
Serum levels of TNF-α, IL-6, and COX-2 and the expression levels of *TNF-α*, *MCP-1*, and *iNOS* mRNA in the colonic epithelium. (**A**) The serum concentrations of TNF-α, IL-6, and COX-2 were measured by enzyme immunoassay; (**B**) The expression levels of *TNF-α*, *MCP-1*, and *iNOS* mRNA in the colonic epithelium were examined by quantitative real-time RT-PCR using specific primers. The values are expressed as mean ± SD. * *p* < 0.01, ** *p* < 0.05.

Chronic inflammation plays a critical role in the pathogenesis of Mets and CRC development [5,8]. Therefore, the levels of inflammatory mediators, including tumor necrosis factor (TNF)-α, interleukin (IL)-6, monocyte chemoattractant protein (MCP)-1, inducible nitric oxide synthase (iNOS), and cyclooxygenase (COX)-2 in hypertensive SHRSP and SHRSP-ZF rats were next examined. The serum levels of TNF-α and IL-6 in SHRSP-ZF rats were significantly elevated compared to those in WKY rats (Figure 3A; *p* < 0.01). The serum levels of COX-2 were also significantly increased in both SHRSP and SHRSP-ZF rats (Figure 3A; *p* < 0.01). In the colonic epithelium of SHRSP-ZF rats, there was a marked increase in the expression of *TNF-α*, *MCP-1*, and *iNOS* mRNA (Figure 3B; *p* < 0.05 compared to WKY rats). Compared to the WKY rats, the expression of *TNF-α* mRNA in the colonic epithelium of SHRSP rats was also significantly increased (Figure 3B; *p* < 0.05).

### 2.6. Discussion

Increasing evidence suggests that Mets is involved in the development of CRC, and this continues to be a growing health problem worldwide, especially in developed countries [1–5]. Recent epidemiological studies have suggested that patients with hypertension, a component of Mets [1,2], comprise a high-risk group for CRC [3–5]. However, appropriate animal models to evaluate hypertension-related colorectal carcinogenesis have not yet been generated.

To our knowledge, the present study provides the first evidence that after administration of AOM, SHRSP and SHRSP-ZF rats, both of which present with hypertension, more readily develop colonic preneoplastic lesions than normotensive WKY rats. In particular, we found that SHRSP rats experience accelerated development of ACF. This is significant because these rats did not exhibit insulin resistance, hyperleptinemia, or dyslipidemia and did not have increased adipose tissue, which are involved in the pathophysiology thought to link Mets to CRC [5–8]. These findings, therefore, suggest that hypertension *per se* might play a critical role in the early events of colorectal carcinogenesis. We have found that the angiotensin converting enzyme inhibitor captopril, an anti-hypertensive drug, significantly prevents the development of ACF in SHRSP-ZF rats [19]. These findings also support our hypothesis that blood pressure elevation *per se* might be directly involved in the early stage of colorectal carcinogenesis. However, in order to test this hypothesis, further studies are needed to establish whether other anti-hypertensive agents, such as AT-II type 1 receptor blockers and calcium channel blockers, can suppress the development of ACF by lowering blood pressure.

Among the pathophysiological disorders associated with hypertension, an increased level of oxidative stress is thought to be particularly important in CRC development [5,6]. Oxidative stress, defined as the overproduction of oxygen species combined with inadequate anti-oxidative defense mechanisms, can result in DNA damage and, consequently, mutations associated with colorectal carcinogenesis [5,20]. In the present study, the hypertensive SHRSP and SHRSP-ZF rats had significantly elevated urine 8-OHdG levels and serum d-ROM levels, which are associated with increased oxidative stress [21]. However, they also had reduced *GPx* and *CAT* mRNA levels, both of which encode antioxidant enzymes, in the colonic epithelium. These findings indicate that both SHRSP and SHRSP-ZF rats are subjected to strong oxidative stress, which might contribute to the development of ACF.

In addition to oxidative stress, the induction of chronic inflammation is also considered to play a critical role in obesity-, diabetes-, and hypertension-related colorectal carcinogenesis [5,6]. In the present study, serum levels of TNF-α and IL-6, as well as colonic expression of *MCP-1* and *iNOS* mRNA, were markedly elevated in SHRSP-ZF obese and diabetic rats. These changes might have been associated with the increase in adipose tissue in SHRSP-ZF rats because excess adipose tissue plays an important role in the exacerbation of systemic inflammation [22,23]. Furthermore, colonic epithelial expression of *TNF-*α mRNA and serum levels of COX-2 were significantly higher in both the hypertensive SHRSP and SHRSP-ZF rats, although the former did not become obese or develop diabetes. These findings are also significant because the dysregulation of TNF-α, a central mediator of chronic inflammatory diseases, and COX-2 have key roles in the stimulation of tumor growth and the progression of carcinogenesis in several tissues, including the colon and rectum [24,25].

Why did the SHRSP rats, which did not exhibit obesity and insulin resistance, experience an acceleration of oxidative stress, exacerbation of chronic inflammation, and development of ACF to the same extent as SHRSP-ZF rats that are both obese and diabetic? One possible explanation is that the dose of AOM (20 mg/kg body weight) used in the present protocol was considerably greater than that needed to induce ACF development in these hypertensive rats. A lower dose of AOM may therefore result in differences in both the number and size of ACF between SHRSP and SHRSP-ZF rats. It is also possible that an increase in the serum level of AT-II, which is the main effector peptide of the renin-angiotensin system [12,13], might contribute to these phenomena because renin-angiotensin system activation has been implicated in the increase in oxidative stress and the induction of inflammation [11,14,26,27]. Renin-angiotensin system activation induces adipocyte inflammation, as demonstrated by the increased expression of TNF-α and IL-6 in adipose tissue, which in turn is implicated in hypertension [28,29]. In prostate cancer, treatment with AT-II stimulates the secretion of IL-6 and MCP-1 from prostate stromal cells and is associated with the increased proliferation of prostate cancer cells [30]. AT-II also induces the expression of iNOS, an inflammatory marker, along with 8-OHdG in prostate cancer cells [31], suggesting a crosslink between renin-angiotensin system-related inflammation and oxidative stress in cancer tissue.

To date, there is no definitive evidence demonstrating the effectiveness of renin-angiotensin system inhibitors in preventing human malignancies, including CRC, in hypertensive patients [32–35]. However, our findings suggest that targeting hypertension-related metabolic abnormalities, including oxidative stress and chronic inflammation caused by renin-angiotensin system activation, may be an effective strategy to prevent CRC development in patients with Mets, especially those with hypertension. In malignant tissue such as CRC, dysregulation of the renin-angiotensin system is implicated in cancer cell migration, invasion, and metastasis [10,11,13,14,36]. A recent study also showed that treatment with renin-angiotensin system inhibitors could inhibit chemically induced colorectal carcinogenesis in obese and diabetic mice by attenuating chronic inflammation and oxidative stress [37]. In order to test the potential efficacy of renin-angiotensin system inhibitors in preventing CRC development in patients with Mets, additional long-term experiments to evaluate whether these agents can prevent colorectal carcinogenesis in hypertensive rats should be conducted.

## 3. Experimental Section

### 3.1. Animals and Chemicals

Five-week-old male SHRSP, SHRSP-ZF, and WKY rats were obtained from Japan SLC (Shizuoka, Japan) and humanely maintained at Gifu University Life Science Research Center in accordance with the Institutional Animal Care Guidelines. The WKY rats are normotensive and not prone to obesity, and thus served as the control group in this study. AOM, which is widely used to mimic sporadic colon carcinogenesis by causing DNA mutations and activating several oncogenic pathways, including the K-*ras* pathway [38,39], was purchased from Wako (Osaka, Japan).

### 3.2. Experimental Procedure

After 1 week of acclimatization, the 6-week-old rats were divided into 3 groups of 8 rats each. All rats received an intraperitoneal injection of AOM (20 mg/kg body weight) once a week for 2 weeks. The experimental protocol and dose of AOM were based on previous studies using F344, Sprague-Dawley, or Wister rat strains [40,41]. We did not include non-AOM treated WKY rats as negative controls because no ACF was found to develop in these animals in a preliminary experiment. At the end of the experiment (2 weeks after the last injection of AOM), when the rats were 10 weeks of age, systolic and diastolic blood pressures were measured noninvasively using a tail cuff (SOFTRON BP98A; Softron, Tokyo, Japan). All rats were euthanized by CO_2_ asphyxiation for colon resection. The third portion of the excised colons (cecum side) was used to extract RNA, and the remaining part was used to determine the number of ACF [42].

### 3.3. Enumeration of ACF

The frequency of AOM-induced colonic premalignant lesions, ACF, was determined as previously described [42]. Briefly, the colon samples were fixed with 10% buffered formalin, stained with methylene blue (0.5% in distilled water) for 20 s, and then placed on microscope slides to count the number of ACF. The number of ACF was recorded along with the number of ACs in each focus. The data are expressed per unit area (cm^2^).

### 3.4. RNA Extraction and Quantitative Real-Time Reverse Transcription-Polymerase Chain Reaction Analysis

The epithelial crypts were isolated from colonic tissue [41]. Total RNA was then extracted from the isolated epithelial crypts using the RNAqueous-4PCR kit (Ambion Applied Biosystems, Austin, TX, USA). cDNA was amplified from 0.2 μg of total RNA using the SuperScript III First-Strand Synthesis System (Invitrogen, Carlsbad, CA, USA). Quantitative real-time reverse transcription-PCR (RT-PCR) analysis was performed using specific primers that amplify *TNF-*α, *MCP-1*, *iNOS*, *GPx*, *CAT*, and glyceraldehyde-3-phosphate dehydrogenase (*GAPDH*) genes. The sequences of these primers, which were obtained from Primer-BLAST [43], are listed in Table S1. Each sample was analyzed on a LightCycler Nano (Roche Diagnostics, Basel, Switzerland) using FastStart Essential DNA Green Master (Roche Diagnostics). Parallel amplification of *GAPDH* was used as the internal control.

### 3.5. Clinical Biochemistry

Blood samples from the inferior vena cava were used for chemical analyses. These samples were obtained at the time of euthanasia, prior to which the rats had fasted for 6 h. The serum levels of TNF-α (R&D Systems, Minneapolis, MN, USA), IL-6 (R & D Systems), insulin (Shibayagi, Gunma, Japan), glucose (BioVision Research Products, Mountain View, CA, USA), leptin (Shibayagi), triglyceride (Wako), NEFA (Wako), AT-II (Phoenix Pharmaceuticals, INC, Burlingame, CA, USA), and COX-2 (MyBioSource, San Diego, CA, USA) were determined using an enzyme-linked immunosorbent assay (ELISA) kit according to the manufacturer instructions.

### 3.6. Oxidative Stress Analysis

Urine 8-OHdG levels were determined using an ELISA kit (NIKKEN SEIL, Shizuoka, Japan). Serum levels of hydroperoxide, a marker for oxidative stress, were evaluated using the d-ROM test (FREE Carpe Diem; Diacron s.r.l., Grosseto, Italy) [21].

### 3.7. Statistical Analysis

All data are presented as mean ± SD and were analyzed using the GraphPad InStat software program version 3.05 (GraphPad Software, San Diego, CA, USA) for Macintosh. One-way analysis of variance (ANOVA) was used to compare groups. If the ANOVA analysis indicated significant differences, the Tukey-Kramer multiple comparisons test was performed to compare the mean values among the groups. The differences were considered significant when the two-sided *p* value was less than 0.05.

## 4. Conclusions

The results of this study indicate that the development of AOM-induced colonic preneoplastic lesions was significantly accelerated in hypertensive rats compared to normotensive rats. This was associated with hypertension-related renin-angiotensin system activation and subsequent induction of oxidative stress and inflammation, suggesting that hypertension plays a critical role in the early stages of CRC.

## Supplementary Information



## Figures and Tables

**Table 1:** Body, liver and adipose weights, BMI and blood pressure of rats.

Group NO.	Strain	No.	Body weight (g)	Relative adipose tissue weight (g/100g body weight) a	Blood pressure (mmHg)	

					Systolic	Diastolic
Group 1	WKY b	8	256.5 ± 11.7 e	0.72 ± 0.16	127 ± 12.8	92 ± 4.9
Group 2	SHRSP c	8	218.9 ± 8.0 f	0.77 ± 0.16	188 ± 12.5 f	141 ± 10.6 f
Group 3	SHRSP-ZF d	8	270.1 ± 23.4 g	1.64 ± 0.17 f,g	169 ± 13.7 f,g	129 ± 9.0 f,g

a White adipose tissue of the periorchis;

b Wister Kyoto/Izm;

c stroke-prone spontaneously hypertensive/Izm;

d SHRSP.Z-*Leprfa*/IzmDmcr;

e Mean ± SD;

f Significantly different from group 1 by Tukey-Kramer Multiple Comparison Test (*p* < 0.001);

g Significantly different from group 2 by Tukey-Kramer Multiple Comparison Test (*p* < 0.05).

**Table 2:** Serum parameters of the experimental rats.

	Glucose (mg/dL)	Insulin (μIU/mL)	Quicki	Leptin (pg/mL)	NEFA (mEq/L)	Triglyceride (mg/dL)	Angiotensin II (ng/mL)
Group 1	85.4 ± 11.7	15.81 ± 0.35	0.313 ± 0.010	11.2 ± 3.6	0.459 ± 0.03	27.1 ± 7.4	352.6 ± 38.1
Group 2	83.5 ± 12.3	17.00 ± 1.39	0.320 ± 0.008	12.2 ± 3.4	0.419 ± 0.05	39.6 ± 14.1	494.4 ± 75.6 b
Group 3	120.0 ± 14.2 b,c	25.60 ± 8.98 b,c	0.291 ± 0.010 b,c	102.7 ± 30.6 b,c	0.538 ± 0.03 b,c	257.1 ± 79.4 b,c	500.9 ± 42.5 b

a Mean ± SD;

b Significantly different from group 1 by Tukey-Kramer Multiple Comparison Test (*p* < 0.05);

c Significantly different from group 2 by Tukey-Kramer Multiple Comparison Test (*p* < 0.05).
