# Supplementary material for: Enhanced Development of Azoxymethane-Induced Colonic Preneoplastic Lesions in Hypertensive Rats
Source: Int J Mol Sci. 2013 Jul 15;14(7):14700–11. doi: 10.3390/ijms140714700 (PMC3742268; doi:10.3390/ijms140714700)
Supplement: Supplementary file 1 [file ijms-14-14700-s001.pdf]

Supplementary Information

Table S1. Primers sequences.

Target gene	Direction	Primer sequence (5'–3')
TNF-α	forward	AACACACGAGACGCTGAAGT
	reverse	TCCAGTGAGTTCCGAAAGCC
MCP-1	forward	TGGGCCTGTTGTTCACAGTT
	reverse	ACCTGCTGCTGGTGATTCTC
iNOS	forward	GTGGTGACAAGCACATTTGG
	reverse	GGCTGGACTTTTCACTCTGC
GPx	forward	TCCACCGTGTATGCCTTCTCC
	reverse	CCTGCTGTATCTGCGCACTGGA
CAT	forward	GAGGCAGTGTACTGCAAGTTCC
	reverse	GGGACAGTTCACAGGTATCTGC
GAPDH	forward	CCTTCATTGACCTCAACTACATGGT
	reverse	TCATTGTCATACCAGGAAATGAGCT

© 2013 by the authors; licensee MDPI, Basel, Switzerland. This article is an open access article distributed under the terms and conditions of the Creative Commons Attribution license (http://creativecommons.org/licenses/by/3.0/).
